# Supplementary material for: The genome sequence of the Small Argent and Sable moth, Epirrhoe tristata (Linnaeus, 1758)
Source: Wellcome Open Res. 2024 Sep 20;9:541. [Version 1] doi: 10.12688/wellcomeopenres.23055.1 (PMC12485329; doi:10.12688/wellcomeopenres.23055.1)
Supplement: Peer review history

# Reviewer report: Reviewer response for version 1

**DOI:** 10.21956/wellcomeopenres.25391.r132276

**Published:** 2025-09-30

**Authors:** Sophie Tandonnet, Alba Almazán

**Affiliations:** 1 Departament de Genetica Microbiologia i Estadistica, Universitat de Barcelona Facultat de Biologia (Ringgold ID: 98709), Barcelona, Catalonia, Spain; 2 Universitat de Barcelona Institut de Recerca de la Biodiversitat (Ringgold ID: 535620), Barcelona, Catalonia, Spain

Summary:

 In this genome note, the authors report the chromosomal-scale assembly of the genome of *Epirrhoe tristata, *the Small Argent and Sable moth. The assembly originates from a single male (the homogametic sex) and contains 30 pseudo-chromosomal molecules (including the Z chromosome) and 2 additional scaffolds. 

 The assembly is of high quality (QV 69.4), completeness (98.4% complete BUSCOs, 0% Ns), highly continuous (most of the genome in chromosome-scale scaffolds) and clean (almost no contamination). The assembly is based on Pacbio HiFi long reads and scaffolded into pseudo-chromosomal molecules using Hi-C data. The same individual (ilEpiTris1) was used for the Pacbio HiFi sequencing (thorax), the HiC (thorax) and the RNA-seq (abdomen), which is a strength of this work.

 The assembly has a corresponding annotation, which we could download without problem. This annotation includes 16,469 genes. The non-coding genes were not annotated. 

 The paper is straightforward and easy to follow. 

 All in all, this note reports a new, high-quality and high-continuity genome assembly of a species not yet sequenced, along with its corresponding annotation. 

 Main Comments:

- How was the specimen's sex determined? Maybe add this to the report.
- Is the picture of the specimen sequenced available? If so, it could be included. 
- Figure 5: it would be useful to indicate which scaffold corresponds to the Z chromosome.
- The authors report 29 autosomes + 1 Z chromosome + 2 other scaffolds.. Table 3 shows 29 autosomes, Z, and the mitochondrial genome, but that adds up to 31. Could the authors clarify what the 32nd scaffold is? If it is an unplaced nuclear scaffold, please list it explicitly in Table 3 for clarity.
- Methods, Nucleic acid extraction: The authors do not explain how they separated the thorax material for HMW DNA extraction and for HiC. Please add those details
- Methods, library preparation: which restriction enzyme was used?
- Methods, Assembly curation: the authors state that they identified the Z chromosome by synteny (to Xanthorhoe spadicearia, as stated in the report section): How conserved is the macrosynteny between E. tristata and X. spadicearia? Is there a clear 1 to 1 chromosomal correspondence? (there must some difference as the chromosome number of both species doesn't seem to be the same). Maybe the authors could show the pattern of synteny

Minor comments

- Background: this section is extremely short; do we know the range of this species? Its basic ecology/lifestyle? 
- It is usually better to sequence the heterogametic sex, although we understand it is not possible to control the individuals you collect.

- The genome annotation availability link ( https://rapid.ensembl.org/Epirrhoe_tristata_GCA_951394285.1/Info/Index ) redirects to: https://beta.ensembl.org/species/81c60124-c548-4d22-96d6-bc1351994b2c

- As far as we could check, all the other links seem to work.
- The page https://tolqc.cog.sanger.ac.uk/darwin/insects/Epirrhoe_tristata/ , compiles metadata and sequencing runs. All seems correct, however, why is there a row with "**OTHER_SOMATIC_ANIMAL_TISSUE**" in the specimen table.
- Methods: Nucleic acid extraction - “ In sample preparation, the ilEpiChri1 sample…” double-check the sample code. Elsewhere it is consistently ilEpiTris1

Are sufficient details of methods and materials provided to allow replication by others?

Partly

Is the rationale for creating the dataset(s) clearly described?

Yes

Are the datasets clearly presented in a useable and accessible format?

Yes

Are the protocols appropriate and is the work technically sound?

Yes

Reviewer Expertise:

Evolutionary biology, Bioinformatics, Non-model organisms, Developmental Biology

We confirm that we have read this submission and believe that we have an appropriate level of expertise to confirm that it is of an acceptable scientific standard.

# Reviewer report: Reviewer response for version 1

**DOI:** 10.21956/wellcomeopenres.25391.r132277

**Published:** 2025-09-29

**Authors:** Erin Toffelmier

**Affiliations:** 1 University of California, Los Angeles, USA

This article describes a genome assembly for *Epirrhoe tristate* as part of the Darwin Tree of Life Project. The article clearly describes the methods and the resulting high quality genome assembly.

 I suggest adding assembly metrics for haplotype 2, even if it’s less complete. It would also be informative to know the expected genome size and karyotype. While this is a very tidy dataset showing strong chromosome-scale scaffolding, with very few unplaced fragments, it would be beneficial to see supporting karyotypic evidence, if available. Finally, consider adding a brief rationale for using *Xanthorhoe spadicearia* in the Z-chromosome synteny analysis (i.e. it’s in the same family).

Are sufficient details of methods and materials provided to allow replication by others?

Yes

Is the rationale for creating the dataset(s) clearly described?

Yes

Are the datasets clearly presented in a useable and accessible format?

Yes

Are the protocols appropriate and is the work technically sound?

Yes

Reviewer Expertise:

Genomics, population genomics, ecology

I confirm that I have read this submission and believe that I have an appropriate level of expertise to confirm that it is of an acceptable scientific standard.
